# Outcomes Comparison Between Primary and Revisional Duodenal Switch in Patients with a BMI Greater than 55 kg/m^2^

**DOI:** 10.3390/jcm14103426

**Published:** 2025-05-14

**Authors:** Lorna A. Evans, Jorge Cornejo, Enrique F. Elli

**Affiliations:** Department of Surgery, Division of Advanced GI and Bariatric Surgery, Mayo Clinic Florida, 4500 San Pablo Rd. S, Jacksonville, FL 32224, USA

**Keywords:** duodenal switch, biliopancreatic diversion with duodenal switch, Single-Anastomosis Duodeno-Ileal Bypass, SADI-s, revisional metabolic bariatric surgery, metabolic bariatric surgery, minimally invasive surgery, robotic-assisted surgery

## Abstract

**Background**: Revisional bariatric surgery for recurrent weight gain is becoming more common, though it carries higher risks and may be less effective than primary bariatric surgery. This study compares clinical outcomes between primary and revisional duodenal switch (DS) in patients with a body mass index (BMI) > 55 kg/m^2^. **Methods**: A retrospective cohort study was conducted on 20 patients who underwent either primary or revisional duodenal switch (DS) surgeries, including biliopancreatic diversion with duodenal switch (BPDDS) and Single-Anastomosis Duodeno-Ileal Bypass with Sleeve Gastrectomy (SADI-s), between January 2015 and December 2023. Revisional DS was defined as the conversion from Sleeve Gastrectomy (SG) to either BPDDS (C-BPDDS) or SADI-S (C-SADI-S). Perioperative and postoperative variables were analyzed. A statistical analysis was performed using chi-square and McNemar tests for categorical variables and Student’s *t*-test for continuous variables. A *p*-value of <0.05 was considered significant. **Results**: Eleven primary DS patients (six BPDDS, five SADI-s) and nine revisional DS patients (five C-BPDDS, four C-SADI-s) were included. The revisional group had a slightly higher preoperative BMI (57.56 ± 5.92 kg/m^2^ vs. 55.93 kg/m^2^ ± 6.91 kg/m^2^). Although operative times were shorter in the revisional group (153.20 ± 53.26 vs. 193.27 ± 46.79 min), the length of stay was longer (2.70 ± 1.25 vs. 2.18 ± 1.16 days). Primary DS patients experienced three minor late complications (dehydration, nephrolithiasis), whereas the revisional group had one major complication (internal hernia requiring reoperation). At the 12-month follow-up, both groups demonstrated similar outcomes in terms of percentage of total weight loss (%TWL) (primary DS: 25.25% ± 12.38 vs. revisional DS: 30.31% ± 10.79) and percentage of excess weight loss (%EWL) (primary DS: 48.41% ± 22.93 vs. revisional DS: 53.24% ± 14.48). **Conclusions**: Revisional DS was associated with shorter operation times and similar weight loss to primary DS. Additionally, it was accomplished safely and led to adequate and sustained weight loss in patients with a BMI greater than 55 kg/m^2^.

## 1. Introduction

Revisional metabolic and bariatric surgery (RMBS) to manage recurrent weight gain after initial weight loss procedures is increasing [1]. Duodenal switch (DS) is gaining recognition as a promising option for patients with severe obesity and is considered one of the most effective bariatric procedures in terms of long-term weight loss and the resolution of obesity-related comorbidities [2,3,4,5]. This approach combines both restrictive and malabsorptive elements, and it is typically recommended for individuals with a BMI over 50 kg/m^2^ [2,3,6,7].

While DS provides a potential solution for managing weight recurrence and obesity-related comorbidities, it is often associated with technical complexities and potential perioperative complications when compared to primary bariatric surgeries [1,8,9].

Primarily due to the complexity of working with previously altered anatomy [6,10,11]. The presence of adhesions and scarring from prior surgeries complicates the dissection and reconstruction process, increasing the risk of organ injury, bleeding, and longer operative times [8]. Additionally, the reduced availability of healthy tissue in previously operated regions of the stomach and small bowel can make the procedure technically more demanding. As a result, perioperative complications such as infections, anastomotic leaks, and internal hernias, as well as higher rates of readmissions and reoperations, are more common in revisional cases [11,12]. Patients undergoing revisional DS often experience longer hospital stays and may face a more prolonged recovery period [8,10]. Moreover, revisional surgeries require more experienced surgical teams and specialized centers due to the increased complexity and resource demands [6].

Nevertheless, there is a growing trend toward using DS as a revisional procedure for patients who have not achieved satisfactory outcomes from previous surgeries, reflecting its feasibility and efficacy in addressing weight recurrence [13,14,15]. Recent data from the American Society for Metabolic and Bariatric Surgery (ASMBS) indicate a steady increase in the use of duodenal switch procedures, particularly the biliopancreatic diversion with duodenal switch (BPDDS), which rose from 1422 cases in 2011 to 6100 cases in 2022 [13]. Similarly, revisional procedures have increased markedly, from approximately 9480 in 2011 to over 31,000 in 2022 [13]. In 2022 alone, BPDDS accounted for 2.2% and SADI-s for 0.6% of all primary bariatric procedures performed in the U.S [16]. These trends suggest that, despite its technical complexity and higher risk profile, DS is increasingly being adopted in clinical practice, due to its safety, feasibility, and potential for durable weight loss and comorbidity resolution [16,17].

This study aimed to compare the clinical outcomes of primary and revisional DS surgeries specifically in patients with a body mass index (BMI) greater than 55 kg/m^2^. Additionally, it will evaluate whether the notion that revisional DS procedures are associated with higher risks and lower effectiveness compared to primary bariatric surgeries can be challenged. By focusing on this distinct patient demographic, we aimed to provide critical insights into the safety and efficacy of these surgical interventions, ultimately aiding the multidisciplinary team in making informed treatment decisions for optimal patient management.

## 2. Materials and Methods

This study was a retrospective cohort study involving twenty patients who underwent either primary or revisional duodenal switch (DS) procedures at our institution from January 2015 to December 2023. The primary procedures included biliopancreatic diversion with duodenal switch (BPDDS) and Single-Anastomosis Duodeno-Ileal Bypass with Sleeve Gastrectomy (SADI-s). Revisional duodenal switch was defined as the conversion from Sleeve Gastrectomy (SG) to either BPDDS (C-BPDDS) or SADI (C-SADI-s).

### 2.1. Inclusion and Exclusion Criteria

The inclusion criteria were as follows: (1) patient’s age ≥ 18 years; (2) patients with a BMI greater than 55 kg/m^2^ who had not undergone any surgical procedures and were considered suitable candidates for a SADI-s or BPDDS as well as patients who underwent SG as a primary procedure and experienced recurrent weight gain after surgery and had obesity-related comorbidities such as diabetes, hypertension, obstructive sleep apnea, and hyperlipidemia; (3) patients who underwent laparoscopic or robotic-assisted approach for primary or revisional BPDDS or SADI-s surgery; (4) complete follow-up data after surgery. The exclusion criteria were as follows: (1) patients who underwent Roux-en-Y gastric bypass as their primary procedure and those who had an adjustable gastric banding as their primary intervention; (2) patients who underwent open BPDDS or SADI-s surgery.

### 2.2. Multidisciplinary Evaluation for Eligibility

The decision to perform revisional surgery was made following a thorough evaluation by a multidisciplinary team, including bariatric surgeons, endocrinologists, and other specialists such as psychologists and nutritionists. This assessment considered factors such as the patient’s clinical status, failure to achieve or maintain weight loss, and persistence of obesity-related comorbidities. The multidisciplinary team also evaluated the patient’s ability to undergo a more complex procedure, factoring in their overall health, nutritional status, and psychological readiness for further surgery.

### 2.3. Statistical Analysis

Patient data included demographics, BMI, past medical and surgical history, including details about previous intervention, operative course, peri-operative outcomes, and follow-up data. Surgical outcomes included the total operative time, intra-operative complications, length of hospital stay (LOS), 30-day post-operative complications, readmissions, and reinterventions. Post-operative outcomes were evaluated in relation to weight status and comorbidity status. The assessment of comorbidities focused on the persistence or resolution of obesity-related conditions, as well as medication use at the last follow-up. Additionally, weight status evaluation included an analysis of weight loss and whether weight recurrence occurred.

Categorical variables were listed as frequencies and numbers (percentages), and group comparisons were performed using the chi-square test for comparisons between two independent groups, or McNemar’s test for paired categorical data. Continuous variables were expressed as means ± standard deviation (SD) values and were compared using the independent Student’s *t*-test for normally distributed data. Bivariate analysis was performed to evaluate patient, operative, and post-operative factors associated with the choice of surgical approach and the symptom outcomes. *p*-values were calculated using two-tailed tests, and a *p*-value of <0.05 was considered significant for all the statistical results. Statistical analyses were performed using the SPSS software program, version 29 (IBM Corp., Armonk, NY, USA).

## 3. Results

### 3.1. Preoperative Analysis

The entire cohort consisted of twenty patients who underwent minimally invasive robotic-assisted surgery. Eleven patients (six BPPDS; five SADI-s) and nine patients (five C-BPDDS; four C-SADI-s) were divided into primary (PG) and revisional (RG) groups. The analysis of preoperative patient demographics and clinical characteristics revealed notable differences between the PG and RG. In terms of gender distribution, the PG comprised 63.6% males and 36.4% females, while the RG consisted of 11.1% males and 88.9% females (Table 1). These results highlight a significant gender imbalance between the two groups at the time of surgery, with a statistically significant difference (*p* = 0.017).

Regarding comorbidities, obstructive sleep apnea (OSA) was present in 90.9% of the PG and 66.7% of the RG. The use of continuous positive airway pressure (CPAP), and bilevel positive airway pressure (BiPAP) devices showed a statistically significant difference, with 81.8% of the PG using these devices compared to 33.3% of the RG (*p* = 0.028) suggesting the more frequent use of these devices for OSA management in the PG.

Lastly, for gastroesophageal reflux disease (GERD), 81.8% of the PG and 77.8% of the RG reported experiencing GERD symptoms, with a *p*-value of 0.822 indicating no significant difference between the groups. Similarly, both groups had the same percentage of patients using proton pump inhibitors (PPIs) or histamine H2 blockers (H2) (*p*-value = 0.436). Further details on clinical characteristics and comorbidities are presented in Table 1.

In terms of preoperative weight, BMI, and age at the time of the surgery, our analysis shows that the mean age at the time of DS for the PG was 38.27 ± 5.64 years, while the RG had a mean age of 45.90 ± 9.29 years with a *p*-value of 0.018, reflecting a notable age difference between groups at the time of surgery (Table 2).

The PG had a mean preoperative weight of 175.09 ± 29.15 kg, compared to 156.07 ± 15.02 kg in the RG (*p*-value = 0.224). For preoperative BMI, the PG had a mean of 55.83 ± 6.91, while the RG had a mean of 57.18 ± 6.15 with a *p*-value of 0.327. Additional details are presented in Table 2.

### 3.2. Intraoperative and Postoperative Analysis

For operational time, the PG had a mean of 193.27 min with an SD of 46.80 min, whereas the RG exhibited a shorter mean operational time of 146.56 min, with an SD of 51.92 min, with a *p*-value of 0.049 (Table 2). This indicates a rather interesting difference between the two groups.

Regarding blood loss during procedures, the PG had a mean blood loss of 35.91 mL with an SD of 31.13 mL, whereas the RG showed a significantly lower mean blood loss of 16.11 mL (SD = 8.94). However, the difference in blood loss between the two groups was not statistically significant. In terms of length of stay, the PG had a mean LOS of 2.18 days (SD = 1.17), while the RG had a longer mean LOS of 2.78 days (SD = 1.30). This difference was also not statistically significant.

The analysis of readmission and reoperation rates was conducted to compare the PG and RG, as shown in Table 2.

For readmissions occurring within 30 days, the PG reported that three patients (27.3%) were readmitted, while the RG had no readmissions. One patient presented with epigastric pain, and a CT scan revealed no evidence of a leak, obstruction, or abscess. The patient’s condition improved with pain management and the use of an abdominal binder; she was discharged later that day. Another patient experienced abdominal pain accompanied by nausea, vomiting, and diarrhea. A CT scan revealed no signs of obstruction, leak, induration, or abscess. The patient was treated with intravenous fluids and pain medication and was also discharged the same day. Additionally, the third patient reported rectal pain and bleeding due to an external anal hemorrhoid. The *p*-value for this comparison was 0.089, indicating that the difference in readmission rates between the two groups approached significance but did not reach conventional thresholds (*p* ≤ 0.05).

In terms of readmissions after 30 days, the PG had six patients (54.5%) readmitted, whereas the RG had two patients (22.2%) readmitted. Among the readmitted patients in the PG, two were readmitted due to ureterolithiasis. Additionally, one patient had three separate readmissions: for dehydration, dysfunctional uterine bleeding, and a panic attack. The final patient in the PG was readmitted for nephrolithiasis. These events were presumed to be unrelated to bariatric surgery. The RG had one patient who was readmitted for lower abdominal pain and vaginal discharge unrelated to bariatric surgery. Additionally, another patient experienced right upper quadrant abdominal pain accompanied by nausea. The abdominal ultrasound was normal with no signs of ascites. The patient received intravenous fluids and was discharged the same day.

Regarding reoperations within 30 days, there were no patients in either group who were reoperated. For reoperations after 30 days, the PG had no patients who underwent reoperation, while RG reported two patients (22.2%). One patient was reoperated for an appendectomy and an internal hernia located between the alimentary limb and the biliopancreatic limb, which occurred 16 months after the bariatric surgery. The hernia was reduced, and the gap was closed with non-absorbable polyester sutures. Additionally, another patient in the RG was reoperated for a cholecystectomy.

At the last follow-up, six out of nine (66.7%) patients in the PG experienced resolution of GERD, compared to six out of seven (85.7%) in the RG (Table 3). PPI or H2 blocker use was similar, with three out of nine (33.3%) patients in the PG and two out of six (33.3%) in RG.

Resolution of OSA was achieved in 3 out of 10 patients (30%) in the PG, whereas all 6 patients (100%) in the RG experienced complete resolution. This marked difference between the groups was statistically significant (*p* = 0.010). The use of CPAP or BiPAP devices decreased significantly in the RG, where all three patients (100%) no longer required respiratory support following surgery. In comparison, only two out of nine patients (22.2%) in the PG were able to discontinue the use of these devices (*p* = 0.045). For more details on postoperative comorbidity resolution, please refer to Table 3.

In terms of postoperative outcomes, at the one-month follow-up, there was minimal difference in the mean postoperative BMI between the PG (51.98 ± 6.78 kg/m^2^) and the RG (52.64 ± 5.24 kg/m^2^), with the *p*-value of 0.833 indicating no significant difference between the two groups (Table 4).

At three months, the PG showed a modest decrease to 47.57 ± 7.38 kg/m^2^, compared to 48.66 ± 4.69 kg/m^2^ in the RG (*p* = 0.722), with no significant difference between the groups. This downward trend continued at six months, where the PG reached a mean BMI of 42.33 ± 7.76 kg/m^2^ and the RG 44.87 ± 5.52 kg/m^2^ (*p* = 0.456). By 12 months, both groups had nearly identical BMIs, 39.61 ± 8.83 kg/m^2^ for the PG and 39.57 ± 3.48 kg/m^2^ for the RG (*p* = 0.993). While the PG consistently showed slightly greater reductions, these differences were not statistically significant, suggesting comparable clinical outcomes between the groups in terms of weight loss.

**Figure 1:**
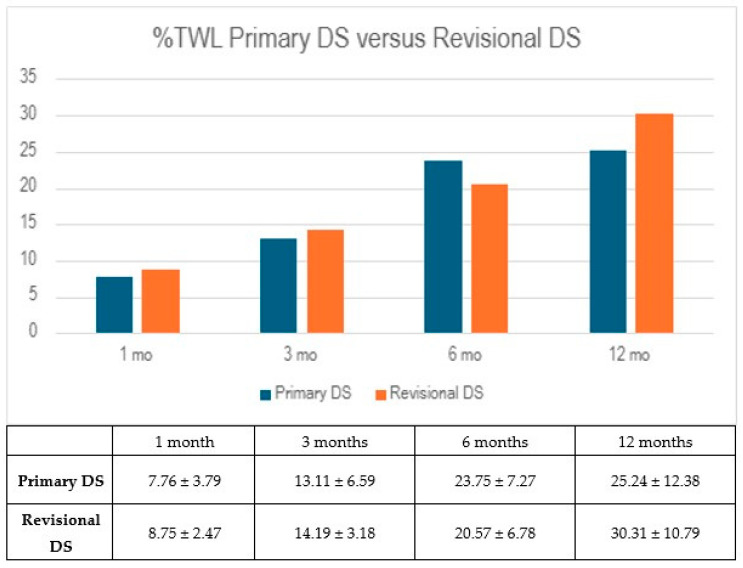
Percentage of total weight loss comparison between primary duodenal switch and revisional duodenal switch. %TWL: percentage of total weight loss; DS: duodenal switch; mo: month/months.

Both groups demonstrated early reductions in body weight, as reflected in their percentage of total weight loss (%TWL). At one month, the RG showed a slightly higher %TWL of 8.75 ± 2.47%, compared to 7.76 ± 3.79% in the PG (*p* = 0.555), though this difference was not statistically significant. By the three-month follow-up, both groups continued to progress, with the PG reaching 13.11 ± 6.59% and the RG 14.19 ± 3.18% (*p* = 0.679). These results suggest similar early weight loss trajectories between the two groups, but the differences did not reach statistical significance (Figure 1).

By six months, the PG showed continued progress in weight reduction, reaching a %TWL of 23.75 ± 7.27%, compared to 20.57 ± 6.78% in the RG (*p* = 0.367). However, at the 12-month mark, this difference reversed slightly, with the RG achieving a higher %TWL of 30.31 ± 10.79%, while the PG reached 25.24 ± 12.38% (*p* = 0.511). Despite these fluctuations, differences between the groups were not statistically significant at either time point.

In examining the percentage of excess weight loss (%EWL), we see similar patterns. At one month, the PG recorded a %EWL of 14.20 ± 6.59%, while the RG reported 15.72 ± 4.42% (*p* = 0.604). At three months, both groups exhibited further improvement, with the PG reaching 24.97 ± 13.29% and the RG 25.58 ± 5.06% (*p* = 0.904). By six months, the PG showed a greater %EWL of 44.46 ± 15.40% compared to 37.36 ± 11.97% in the RG (*p* = 0.310). At the 12-month follow-up, the RG once again demonstrated a higher %EWL of 53.24 ± 14.48%, while the PG reached 48.40 ± 22.93% (*p* = 0.690). Overall, both groups achieved meaningful weight loss over the study period, though intergroup differences did not reach statistical significance.

## 4. Discussion

Our findings reveal significant demographic and clinical differences between the two groups, particularly in gender distribution and age at the time of surgery. The PG comprised a higher percentage of males (63.6%) compared to the RG (11.1%), which may reflect varying motivations and health-seeking behaviors in different populations. This aligns with other studies that have reported gender disparities in bariatric surgery candidates, where men are often more likely to pursue primary interventions, while women more frequently undergo revisional procedures due to earlier surgeries that may not have achieved desired outcomes [9,15,18].

This disparity may also reflect a higher prevalence of weight recurrence in women following primary bariatric surgery. While the underlying causes of weight gain remain incompletely understood, and the literature does not provide a conclusive explanation as to whether women are more likely to seek revisional surgery or the reasons behind this, we hypothesize that biological, hormonal, and behavioral factors could contribute to this finding. For instance, hormonal fluctuations, particularly in estrogen levels during the perimenopausal and postmenopausal phases, may lead to more significant weight fluctuations in women [19,20]. Additionally, psychosocial stress, emotional eating patterns, and differences in body composition and fat distribution may complicate long-term weight maintenance, making it more challenging for women to sustain weight loss [19,20,21]. These factors may therefore increase the likelihood of women seeking revisional surgery when outcomes from primary surgery are not preserved over time.

Although our study did not specifically investigate gender differences in the context of weight recurrence, future research should focus on how hormonal, metabolic, psychosocial, and behavioral factors influence the decision to pursue revision surgery in female patients.

In terms of comorbidities, our analysis revealed comparable resolution rates of hypertension, diabetes, and GERD between both groups, with no statistically significant differences. Several studies have reported similar results in comorbidities resolution after primary DS/SADI-s surgery [2,22,23,24].

Interestingly, hyperlipidemia resolution was achieved in 33.3% of patients in the PG, whereas all the patients in the RG experienced resolution. Although the RG demonstrated numerically higher resolution rates, this difference did not reach statistical significance. One possible explanation for this observed difference is that patients in the RG may have undergone more rigorous dietary counseling and follow-up care after experiencing suboptimal outcomes from their initial procedures, leading to better long-term adherence to lifestyle modifications. Additionally, the physiological changes from undergoing two bariatric surgeries may have resulted in greater metabolic improvements, including lipid profile normalization, compared to those who had only one primary procedure, as reported by Koh et al., who observed the remission of hyperlipidemia following revisional bariatric surgery [25]. Furthermore, several studies in the literature have shown improvements in hyperlipidemia remission or hyperlipidemia reduction for patients who underwent DS/SADI-s surgery [2,22,24,26]. Further studies with larger cohorts are necessary to better understand the potential role of revisional surgery in hyperlipidemia resolution.

OSA was more prevalent in the PG (90.9%) compared to the RG (66.7%). Notably, the resolution of OSA was markedly higher in the RG, with 100% of patients no longer requiring CPAP/BiPAP devices compared to only 22.2% in the PG. This represents a 68.7% reduction in OSA burden in the RG compared to only 20.2% in the PG. These statistically significant findings emphasize the potential benefits of the anatomical and metabolic modifications achieved during revisional DS, which may enhance upper airway stability and reduce apnea severity beyond what is observed with primary operations. The more significant improvement observed in the RG group could be due to a combination of sustained weight loss and better adherence to postoperative lifestyle changes. As mentioned before, these improvements may stem from the enhanced postoperative care that typically accompanies revisional surgeries, which, due to their complexity, require more structured follow-up and closer monitoring.

In summary, while the RG group showed clear improvements in the resolution of OSA and the use of CPAP/BiPAP devices, other comorbidities such as GERD, hyperlipidemia, and hypertension showed numerically higher resolution rates in the RG, though these differences did not reach statistical significance. These findings suggest that the effectiveness of revisional surgery may vary across different comorbidities. For conditions like hyperlipidemia and GERD, resolution may depend more on factors such as medication use, dietary changes, and individual responses to surgery, rather than the type of surgery alone.

Interestingly, our findings are consistent with the previous literature suggesting that bariatric surgeries, including DS, may be more effective in resolving obesity-related comorbidities compared to other bariatric procedures [2,22,24,26].

In regard to intraoperative surgical outcomes, the PG experienced longer surgical times (*p* = 0.049) and greater intraoperative blood loss. This observation contrasts with findings from similar studies, where primary procedures result in shorter operative durations and less blood loss [8,11]. This discrepancy may be attributed to the fact that revisional cases are typically handled by highly skilled and experienced surgeons, while primary surgeries can sometimes be performed by those with varying levels of expertise, given their more straightforward nature. Furthermore, this finding could be linked to the population that seeks care at highly specialized institutions, which are equipped to manage complex cases and often attract patients with more challenging medical histories. These centers not only manage a higher volume of revisional procedures but also have the resources and expertise necessary to ensure optimal results.

Our analysis of readmission and reoperation rates between the PG and RG groups reveals important trends and potential insights into the postoperative management of patients undergoing bariatric surgery. In terms of readmissions within 30 days, the PG group had three patients (27.3%) readmitted, while the RG group had none. Although the *p*-value of 0.089 indicates that this difference is not statistically significant, it suggests a potential trend and raises important considerations about the factors contributing to higher readmission rates in the PG group. Most of these readmissions appeared unrelated to the bariatric procedure itself, including cases of abdominal discomfort with no postoperative complications identified, and one case of rectal pain due to an external hemorrhoid. For readmissions beyond 30 days, the PG group experienced a higher rate, with six patients (54.5%) compared to two patients (22.2%) in the RG group. While this difference is not statistically significant, it may still warrant further research to determine the clinical relevance of the observed differences. Interestingly, our findings contrast with the results reported by Wang et al., who observed higher trends of readmissions in the RG group [8]. Similarly, most readmissions beyond 30 days, particularly in the PG group, were due to conditions such as ureterolithiasis, dehydration, dysfunctional uterine bleeding, panic attack, and nephrolithiasis. Furthermore, in the RG group, one patient was readmitted for lower abdominal pain and vaginal discharge, while another presented with right upper quadrant pain and nausea, neither of which revealed surgical complications on imaging. Although readmissions beyond 30 days were observed, many appeared unrelated to the bariatric procedure itself. These observations highlight the potential value of implementing more structured postoperative follow-up protocols and enhancing patient education, particularly for those undergoing primary bariatric surgery, to help minimize preventable readmissions.

When examining reoperation rates after 30 days, the PG group demonstrated no instances of reoperation, whereas two patients (22.2%) from the RG group required reoperation, which aligns with the trends reported by Wang et al. [8]. One patient underwent an appendectomy, followed by surgical repair of an internal hernia between the alimentary and biliopancreatic limbs, which developed 16 months after BPDDS.

According to recent research, including a multicenter evaluation of 1160 patients undergoing BPDDS and SADI-s, the majority of internal hernias occur within the first 24 months after surgery [27]. In addition, they reported an overall incidence of 1.12%, with the majority happening in the pseudo-Petersen’s space [27]. Abdominal pain was the most common presenting symptom, and a CT scan was the primary diagnostic tool used to assess these patients [27]. Interestingly, in their study, they found that SADI-S was linked to a marginally decreased risk of developing internal hernias when compared to BPDDS [27]. These findings reinforce the importance of considering internal hernia in the differential diagnosis of postoperative abdominal pain and tailoring surveillance strategies accordingly.

While RMBS offers benefits in weight loss and comorbidity resolution, it is a complex procedure that inherently carries a higher risk of certain complications. The presence of reoperations in the RG group, despite a lower overall readmission rate, highlights the multifactorial nature of postoperative outcomes and underscores the need for continued monitoring and long-term follow-up in this patient population.

As observed in our study, postoperative outcomes such as readmission and reoperation rates may be influenced by a variety of factors, underscoring the importance of structured postoperative care. This includes comprehensive nutritional support and strict adherence to follow-up protocols. At our institution, patients are introduced to a staged structured diet progression, starting with clear liquids and advancing to blenderized and mechanical-soft foods, all while ensuring adequate protein intake (60–75 g per day). Adherence to this diet is essential to prevent nutritional deficiencies and complications. Additionally, follow-up visits at regular intervals are vital for monitoring progress, assessing for potential complications, and adjusting the patient’s care plan as necessary. In particular, regular check-ins are crucial and may include monitoring fluid intake, ensuring compliance with vitamin and mineral supplementation, and addressing postoperative issues such as dehydration, pain, or psychological concerns. While adherence to these protocols may significantly improve outcomes, patient compliance can be influenced by factors such as the clarity of preoperative education, access to resources, and ongoing support. A more structured follow-up system, enhanced patient education, and personalized care could further reduce readmissions and improve postoperative recovery, ultimately ensuring the best possible outcomes for bariatric surgery patients.

Regarding weight loss outcomes, both groups showed comparable reductions in BMI, percentage of total weight loss (%TWL), and percentage of excess weight loss (%EWL) at various follow-up points. Specifically, the RG achieved a slightly higher %TWL at 12 months (30.31%) compared to the PG (25.24%), although this difference did not reach statistical significance. In our cohort, the %EWL was similar at one, three, and six months; however, at twelve months, the RG exhibited a slightly higher %EWL (PG 48.40 + 22.93 vs. 53.24 + 14.48 RG).

As mentioned earlier, these findings may further support the notion that more intensive postoperative interventions, including enhanced dietary counseling, structured follow-up, and lifestyle modifications, could contribute to the slightly better long-term outcomes observed in the RG group. Additionally, the metabolic effects associated with undergoing a second bariatric procedure might explain the observed differences in the RG’s weight loss. We also hypothesize that the nature of revisional surgery itself, being a second procedure, could influence patient behavior. Patients who undergo revisional surgery are likely more aware of the limited options for managing their weight. Additionally, they are more cognizant of the complexity and risks associated with such procedures. This increased awareness and understanding of the challenges involved could potentially enhance their commitment to weight loss strategies and adherence to postoperative recommendations.

When compared to other studies that evaluated %TWL and %EWL following primary SADI-s surgery at similar follow-up points, our results for both primary and revisional DS surgery are lower [22,23].

It is important to note that postoperative care plays a critical role in optimizing outcomes after primary bariatric surgery or RMBS, particularly through comprehensive nutritional support and adherence to follow-up protocols. At our institution, patients are introduced to a staged structured diet progression, starting with clear liquids and advancing to blenderized and mechanical-soft foods, all while ensuring adequate protein intake (60–75 g per day). Adherence to this diet is essential to prevent nutritional deficiencies and complications. Additionally, follow-up visits at regular intervals are vital for monitoring progress, assessing for potential complications, and adjusting the patient’s care plan as necessary. In particular, regular check-ins are crucial and may include monitoring fluid intake, ensuring compliance with vitamin and mineral supplementation, and addressing postoperative issues such as dehydration, pain, and psychological concerns. While adherence to these protocols may significantly improve outcomes, patient compliance can be influenced by factors such as the clarity of preoperative education, access to resources, and ongoing support. A more structured follow-up system, enhanced patient education, and personalized care could further reduce readmissions and improve postoperative recovery, ultimately ensuring the best possible outcomes for bariatric surgery patients.

Overall, our findings contribute to the growing body of evidence supporting the feasibility, safety, and effectiveness of DS as a revisional procedure. Similar weight loss outcomes and resolutions of comorbidities suggest that revisional DS can be a viable option for patients whose initial surgical intervention was ineffective in achieving weight loss and resolving obesity-related comorbidities. Further research comparing primary and revisional DS procedures with larger cohorts is essential to better understand their respective outcomes and to optimize patient management strategies.

## 5. Conclusions

Differences in demographics, comorbidity resolution, and surgical outcomes were noted between primary and revisional duodenal switch surgeries. The revisional group exhibited higher rates of comorbidity resolution, particularly for obstructive sleep apnea, while the primary group encountered longer surgical times and higher readmission rates. Despite these distinctions, both groups achieved comparable rates of weight loss and resolution of comorbidities. These findings suggest that revisional duodenal switch is not only feasible but also a safe and effective option for patients with obesity-related comorbidities experiencing recurrent weight gain. Further studies with larger, multicenter cohorts and longer follow-up periods are needed to corroborate these findings and provide a deeper insight into the long-term effects and outcomes.

### Limitations

This study has several limitations. Firstly, as a retrospective analysis with a relatively small sample size, the statistical power of the findings is limited and may restrict the generalizability of the findings. Therefore, the results should be interpreted with caution and regarded as exploratory rather than definitive. The small sample size also precluded the use of statistical adjustments for baseline differences between groups, which may influence the interpretation of the results. Larger cohorts with sufficient statistical power are essential to confirm these findings and allow for meaningful subgroup comparisons. Additionally, such cohorts would strengthen the robustness of the data and potentially uncover additional trends. Secondly, the study primarily concentrated on short-term outcomes, leaving long-term weight loss and comorbidity resolution insufficiently examined. In addition, patient adherence to postoperative recommendations, a critical factor that can significantly affect results, was not assessed. Moreover, all procedures were performed at a high-volume center by a highly experienced surgical team. While this contributes to the consistency and quality of care, it may limit the applicability of the findings to other settings with differing resources or levels of surgical expertise. Future research should aim for larger, multi-center cohorts with extended follow-up periods to yield more definitive conclusions.

## Figures and Tables

**Table 1:** Preoperative demographics and clinical characteristics.

	Preoperative Demographics and Clinical Characteristics		
Variable	Primary DS	Revisional DS	*p*-Value
Male	7/11 (63.60%)	1/9 (11.1%)	**0.017**
Female	4/11 (36.4%)	8/9 (88.9%)	**0.017**
Hiatal Hernia	11/11 (100%)	8/9 (88.9%)	0.257
GERD	9/11 (81.8%)	7/9 (77.8%)	0.822
PPI/H2 Blocker Use	9/11 (81.8%)	6/9 (66.7%)	0.436
Obstructive Sleep Apnea	10/11 (90.9%)	6/9 (66.7%)	0.178
CPAP/BiPAP Use	9/11 (81.8%)	3/9 (33.3%)	**0.028**
Diabetes Mellitus	9/11 (81.8%)	7/9 (77.8%)	0.822
Glucose-Lowering Agents	9/11 (81.8%)	7/9 (77.8%)	0.822
Hypertension	6/11 (54.5%)	4/9 (44.4%)	0.653
Anti-Hypertensives Use	6/11 (54.5%)	5/9 (55.6%)	0.964
Hyperlipidemia	3/11 (27.3%)	3/9 (33.3%)	0.769
Lipid-Lowering Agents	3/11 (27.3%)	3/9 (33.3%)	0.769

DS: duodenal switch; GERD: gastroesophageal reflux disease; PPI/H2 blockers: proton pump inhibitor/histamine blockers; CPAP/BiPap: continuous positive airway pressure/bilevel positive airway pressure. Data are presented as frequency and percentage for categorical variables.

**Table 2:** Comparison of preoperative, intraoperative, and postoperative outcomes between primary and revisional duodenal switch groups.

		Primary DS	Revisional DS	*p*-Value
**Preoperative**				
	Age at DS (years)	38.27 ± 5.64	45.90 ± 9.29	**0.018**
	BMI (kg/m^2^)	55.83 ± 6.91	57.18 ± 6.15	0.327
	Weight (kg)	175.09 ± 29.15	156.07 ± 15.02	0.224
	Time from SG to c-DS (months)	-	62.67 ± 29.26	-
**Intraoperative**				
	OP Time (min)	193.27 ± 46.80	146.56 ± 51.92	**0.049**
	LOS (days)	2.18 ± 1.17	2.78 ± 1.30	0.295
	Blood Loss (mL)	35.91 ± 31.13	16.11 ± 8.94	0.083
**Postoperative**				
	Readmission < 30 days	8/11 (72.7%)	9/9 (100%)	0.089
	Readmission > 30 days	5/11 (45.5%)	7/9 (77.8%)	0.142
	Reoperation < 30 days	0/11 (0%)	0/9 (0%)	-
	Reoperation > 30 days	0/11 (0%)	2/9 (22.2%)	0.099

DS: duodenal switch; BMI: body mass index; SG: Sleeve Gastrectomy; c-DS: conversion to duodenal switch; OP time: operative time; LOS: length of hospital stay.

**Table 3:** Obesity-related comorbidity resolution and medication use of primary versus revisional DS at last follow-up.

	Postoperative Comorbidity Resolution		
Comorbidity	Primary DS	Revisional DS	*p*-Value
GERD	6/9 (66.7%)	6/7 (85.7%)	0.383
PPI/H2 blocker Use	3/9 (33.3%)	2/6 (33.3%)	1.00
Obstructive Sleep Apnea	3/10 (30%)	6/6 (100%)	**0.010**
CPAP/BiPAP Use	2/9 (22.2%)	3/3 (100%)	**0.045**
Diabetes Mellitus	1/2 (50%)	1/2 (50%)	1.000
Glucose-Lowering Agents Use	1/2 (50%)	1/2 (50%)	1.000
Hypertension	2/6 (33.3%)	2/5 (40%)	0.819
Anti-hypertensives Use	2/6 (33.3%)	2/5 (40%)	0.819
Hyperlipidemia	1/3 (33.3%)	3/3 (100%)	0.200
Lipid-Lowering Agents Use	1/3 (33.3%)	3/3 (100%)	0.200

GERD: gastroesophageal reflux disease; PPI/H2 blockers: proton pump inhibitor/histamine blockers; CPAP/BiPAP: continuous positive airway pressure/bilevel positive airway pressure. Data are presented as frequencies and percentages for categorical variables.

**Table 4:** Weight-loss outcomes after primary versus revisional DS.

	Primary DS	Revisional DS	*p*-Value
**Postop BMI (kg/m^2^)**			
1 mo	51.98 ± 6.78	52.64 ± 5.24	0.833
3 mo	47.57 ± 7.38	48.66 ± 4.69	0.722
6 mo	42.33 ± 7.76	44.87 ± 5.52	0.456
12 mo	39.61 ± 8.83	39.57 ± 3.48	0.993
**%TWL**			
1 mo	7.76 ± 3.79	8.75 ± 2.47	0.555
3 mo	13.11 ± 6.59	14.19 ± 3.18	0.679
6 mo	23.75 ± 7.27	20.57 ± 6.78	0.367
12 mo	25.24 ± 12.38	30.31 ± 10.79	0.511
**%EWL**			
1 mo	14.20 ± 6.59	15.72 ± 4.42	0.604
3 mo	24.97 ± 13.29	25.58 ± 5.06	0.904
6 mo	44.46 ± 15.40	37.36 ± 11.97	0.310
12 mo	48.40 ± 22.93	53.24 ± 14.48	0.690

Postop BMI: postoperative BMI; %TWL: percentage of total weight loss; %EWL: percentage of excess weight loss; DS: duodenal switch; mo: month/months.

## Data Availability

The original contributions presented in this study are included in the article. Further inquiries can be directed to the corresponding author.

## Abbreviations

The following abbreviations are used in this manuscript:

RMBS	Revisional metabolic and bariatric surgery
DS	Duodenal switch
BMI	Body mass index
BPDDS	Biliopancreatic diversion with duodenal switch
SADI-s	Single-Anastomosis Duodeno-Ileal Bypass with Sleeve Gastrectomy
SG	Sleeve Gastrectomy
C-BPDDS	Conversion to BPDDS
C-SADI-s	Conversion to SADI
LOS	Length of hospital stay
SD	Standard Deviation
PG	Primary group
RG	Revisional group
OSA	Obstructive sleep apnea
CPAP	Continuous positive airway pressure device
BiPAP	Bilevel positive airway pressure device
GERD	Gastroesophageal reflux disease
PPI	Proton pump inhibitors
H2	Histamine H2 blockers
# pts	Number of patients
% pts	within group Percentage of patients within the group
OP time	Operative time
Postop BMI	Postoperative BMI
%TWL	Percentage of total weight loss
%EWL	Percentage of excess weight loss
mo	Month/months
